# 

**Published:** 1917-08-18

**Authors:** 


### THE WAR RECORD OF ST. THOMAS'S HOSPITAL.

Up to August 8, 1917, the war honours of the 972 past and present students of St. Thomas's Hospital who were serving are 39 killed in action or died on service; one V.C., one K.C.B., two K.C.M.G.s, four C.B.s, eleven C.M.G.s, one M.V.O., sixteen D.S.O.s, twenty-three M.C.s, one Bar to M.C. The names of 101 men have been mentioned in dispatches and for valuable service 146 times. The foreign honours include four French, four Serbian, two Belgian, one Egyptian, and three Orders of the Hospital of St. John of Jerusalem.

Rates of Subscription: Twelve months, 6/6; Six months, 4/-; three months, 2/-, post free to any address in the United Kingdom. Abroad, Twelve months, 8/8; Six months, 5/-; Three months, 2/6, post free. THE HOSPITAL "Index" to Volume LXI., October 1916 to March 1917, is now ready, price 2½d. per copy, post free. Address The Manager, The Hospital, "The Hospital" Building, 28/29 Southampton Street, Strand, London, W.C. 2.

**A NEW TREATMENT FOR GONORRHŒA.**
By CHARLES RUSS, M.B., M.R.C.S.,
*Physician-in-Charge, Electro-Therapeutic Department, Male Lock Hospital, London.*
Demy 8vo, 3s. net; post free, 3s. 4d.

"We have read this book with interest and profit, and cordially recommend it to the profession."—*The Medical Press.*
"This form of treatment merits further attention, as it is both scientific and ingenious."—*British Medical Journal.*

A New Edition is in preparation.

London: H. K. LEWIS & CO., Ltd., 136 Gower Street, W.C.

